# Bi-Level Coordinated Merging of Connected and Automated Vehicles at Roundabouts

**DOI:** 10.3390/s21196533

**Published:** 2021-09-30

**Authors:** A. S. M. Bakibillah, Md Abdus Samad Kamal, Chee Pin Tan, Susilawati Susilawati, Tomohisa Hayakawa, Jun-ichi Imura

**Affiliations:** 1 School of Engineering and Advanced Engineering Platform, Monash University, Bandar Sunway 47500, Malaysia; asm.bakibillah@monash.edu (A.S.M.B.); tan.chee.pin@monash.edu (C.P.T.); susilawati@monash.edu (S.S.); 2 Graduate School of Science and Technology, Gunma University, Kiryu 376-8515, Japan; 3 Department of Systems and Control Engineering, Tokyo Institute of Technology, Tokyo 152-8552, Japan; hayakawa@sc.e.titech.ac.jp (T.H.); imura@sc.e.titech.ac.jp (J.-i.I.)

**Keywords:** bi-level coordination, connected and automated vehicles, combinatorial optimization, roundabout, receding horizon, vehicle clustering

## Abstract

Traditional uncoordinated traffic flows in a roundabout can lead to severe traffic congestion, travel delay, and the increased fuel consumption of vehicles. An interesting way to mitigate this would be through cooperative control of connected and automated vehicles (CAVs). In this paper, we propose a novel solution, which is a roundabout control system (RCS), for CAVs to attain smooth and safe traffic flows. The RCS is essentially a bi-level framework, consisting of higher and lower levels of control, where in the higher level, vehicles in the entry lane approaching the roundabout will be made to form clusters based on traffic flow volume, and in the lower level, the vehicles’ optimal sequences and roundabout merging times are calculated by solving a combinatorial optimization problem using a receding horizon control (RHC) approach. The proposed RCS aims to minimize the total time taken for all approaching vehicles to enter the roundabout, whilst minimally affecting the movement of circulating vehicles. Our developed strategy ensures fast optimization, and can be implemented in real-time. Using microscopic simulations, we demonstrate the effectiveness of the RCS, and compare it to the current traditional roundabout system (TRS) for various traffic flow scenarios. From the results, we can conclude that the proposed RCS produces significant improvement in traffic flow performance, in particular for the average velocity, average fuel consumption, and average travel time in the roundabout.

## 1. Introduction

Traditional human driving behavior, such as lack of anticipatory driving and response to various disturbances, causes severe traffic congestion and accidents. Traffic congestion significantly increases fuel consumption, greenhouse gas (GHG) emissions, and travel delays [1]. In particular, signalized intersections, merging roadways, and roundabouts are the main sources of traffic congestion, with frequent idling, acceleration, and braking [2]. Field studies show that stop-and-go vehicles produce 14% more emissions than vehicles driven at a constant speed [3]. A report on urban road mobility reveals that traffic congestion caused American drivers to travel 8.8 billion hours extra on the road and consume additional 3.3 billion gallons of fuel, resulting in a total cost of 179 billion USD per year [4]. According to the European Commission, the external costs of road traffic congestion alone amount to 0.5% of community GDP, and by 2050, the costs will increase by about 50% [5]. Thus, policymakers and researchers have been focusing on various sustainable road transportation technologies to deal with an increasing number of vehicles and traffic congestion, by utilizing the existing road-traffic paradigm.

Intelligent transportation systems (ITS), which provide a variety of solutions for transportation and traffic management systems, are one promising option for reducing traffic congestion. Specifically, recent connected and automated vehicle (CAV) technologies (infra-vehicle (I2V) and vehicle-vehicle (V2V) communications) enable real-time vehicle information to be available, which can be utilized to precisely control the movement of these vehicles [6]. Furthermore, a centralized or decentralized controller can coordinate vehicle and infrastructure to increase traffic flow performance and safety in certain conditions, such as signalized intersections and merging roadways [7]. The concept of a coordinated traffic system has generated a lot of interest in traffic flow control, since it addresses a variety of challenges that human drivers face, such as stop-and-go driving and traffic accidents. Vehicles in a traffic coordination system are expected to execute the controller’s commands to ensure that traffic flow is as efficient as possible. Furthermore, coordination can be done repeatedly for smooth operation, even if the vehicle does not execute the command, or if unanticipated disruptions occur. Therefore, it is able to precisely reconfigure the entire system, which is extremely difficult for a human driver to do.

Several works have developed vehicle coordination systems for signalized intersections and merging roadways using centralized or decentralized approaches. Some studies focused on the utilization of advanced signal phase and timing (SPAT) information within the adaptive cruise control system of the automated vehicle [8]; whereas some studies proposed the optimization of traffic-light or signal phases based on the state information (position and velocity) of connected vehicles [9]. Some researchers developed a cooperative vehicle intersection control (CVIC) system [10] and a vehicle intersection coordination scheme (VICS) [11], for futuristic autonomous vehicles, without using traffic lights under connected vehicles environment. In [12], a unified framework is developed for the integrated optimization of traffic signals and CAV trajectories for isolated signalized intersections. As an alternative to the traditional traffic control systems, some studies proposed autonomous intersection management (AIM) systems based on reservation algorithms [13]. It was also demonstrated that self-driving data can be effectively used to achieve efficient driving in a signalized intersection [14]. On the other hand, some researchers proposed merging control systems with cooperation between intelligent vehicles and infrastructure to ensure safe maneuvering at road intersections [15]. Some other works demonstrated fuzzy controllers for the smooth merging of vehicles [16], whereas [17] proposed a decentralized control approach to achieve safe merging maneuver. A model predictive control (MPC) framework for cooperative merging was presented in [18]. Several other research efforts have been reported on V2I coordination systems for safe and efficient merging into freeways using ramp metering, such as feedback control systems [19] and optimal control systems [20]. While many works on vehicle coordination at signalized intersections or merging roadways have been proposed, very few works have been reported on traffic coordination at roundabouts.

A roundabout is a specific case of merging intersections or unsignalized roadways, where vehicles merge at low speed for safe interaction with other circulating vehicles, traverse the roundabout, and eventually exit to their desired directions. The operational and safety characteristics of roundabouts are usually better than typical intersections and merging roadways [21], but the yielding vehicles on the entry lane must be aware of vehicles on the circulating lane to avoid collision. Since an approaching vehicle requires an extra gap to enter the roundabout, increased traffic flow at the merging point can have a severe impact on the roundabout capacity. Consequently, the fuel consumption and delay entering the roundabout can increase substantially.

### Related Works

Hummer et al. [22] developed a simple macroscopic roundabout model to observe the effectiveness of metering signals with peak period demands. In [23], the benefits of using metering signals to mitigate operational problems with unbalanced flow at roundabouts are explored. Xu et al. [24] developed a multi-level traffic control (MTC) algorithm that combines hybrid yield with fully actuated control at a large four-leg roundabout to automatically facilitate time-varying vehicular demands. The works [22,23,24] mainly focused on improving mobility and safety at roundabouts during rush hours using ramp metering with traffic signal control. In [25], the concept of virtual platooning is adapted to roundabout crossing, where they used high definition maps with a curvilinear coordinates framework. Rodrigues et al. [26] proposed an adaptive tactical behavior planner (ATBP) for CAVs, which is capable of planning human-like motion behaviors for navigating a non-signalized roundabout. The works [25,26] aimed to achieve roundabout collision avoidance for automated vehicles. In [27], a rule-based roundabout management system was developed for optimal coordination of CAVs to improve its traffic performance. Zhao et al. [28] proposed an optimal roundabout coordination scheme for CAVs, where they examined the effects of varying levels of CAV market penetration. In [29], a cloud-based optimal coordination system is developed for a four-leg roundabout. The works [28,29] developed optimal roundabout coordination strategies based on merely a balanced traffic flow condition. The optimization-based solutions ensure optimal performance in a variety of traffic situations; however, these solutions may not always yield the global optimum solution in the time frame needed for roundabout management. Moreover, the computational complexity of optimization-based solutions grows enormously when the traffic volume and complexity of the scenario rise [30]. Hence, most of the existing optimization-based solutions are not applicable for real-time control.

In this paper, we develop a novel bi-level roundabout control system (RCS), consisting of a higher level and a lower level coordination scheme for CAVs using a centralized controller called roundabout coordination unit (RCU) at a four-leg roundabout. The aim is to maximize traffic flow and minimize idling time via safe maneuvering at merging points for various balanced and unbalanced traffic demands. The higher level of coordination uses traffic flow information to form vehicle clusters (platoons), whilst the lower level coordination solves a receding horizon control optimization problem to determine the optimal merging sequence and trajectory of vehicles. Our proposed successive receding horizon control approach makes the optimization problem simple and real-time implementable for different traffic volume and complexity. We evaluate the effectiveness of the proposed scheme through microscopic traffic simulation for various traffic demands. From the results, it is evident that the proposed RCS reduces average fuel consumption, average travel time, and increases average velocity compared to the traditional human driving.

This paper is organized as follows. Section 2 describes the general idea of our proposed RCS and the modeling of traffic flow. Section 3 formulates the optimization problem, including higher and lower level coordination. Key simulation results are presented in Section 4, and finally, Section 5 provides a discussion and a conclusion for the proposed scheme.

## 2. Roundabout Control Scheme

**Figure 1:**
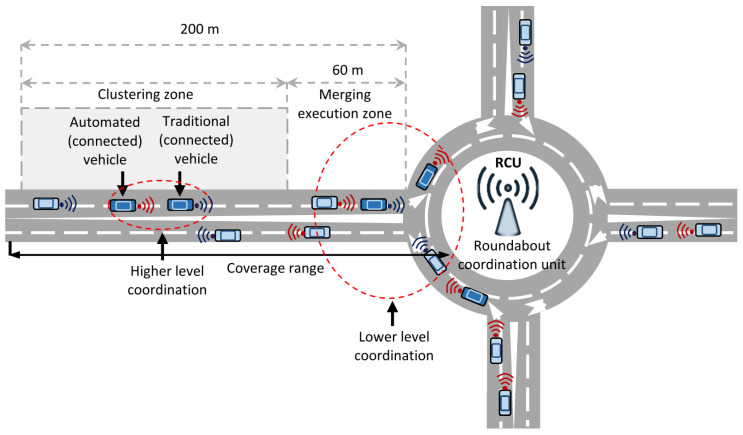
The proposed bi-level roundabout control system (RCS). The environment contains automated and traditional connected vehicles. The RCU has two-way communication facility and coordinates automated vehicles at the higher and lower levels.

Figure 1 illustrates the proposed bi-level roundabout control system in an environment of connected vehicles. In this paper, we consider an unsignalized four-legged single-lane roundabout; the legs are equally spaced at $90^{\circ }$, and each leg has an entry and an exit lane. We consider a roundabout coordination unit that can communicate in two-ways (I2V and V2I), with negligible delay to globally coordinate the vehicles. The full signal coverage range of the RCU is a few hundred meters. The vehicles frequently transmit their information, such as the current position and velocity to the RCU within the coverage range. For simplicity, all vehicles are assumed to be automated; such an assumption is reasonable, as traditional connected vehicles can be considered to comply with speed advice generated by the RCU. Hence, in this paper, all vehicles are assumed to be CAVs.

Specifically, we define two zones for the implementation of the bi-level coordination, namely the *clustering zone* and *merging-execution zone*. As shown in Figure 1, we define the clustering zone as the road segment between 60 m to 200 m from the roundabout merging point, and the merging execution zone is from the merging point to 60 m away (between the merging point and the clustering zone). The RCU then determines if it is necessary to form a vehicle cluster; if necessary, then vehicles in the clustering zone will be directed to form vehicle clusters. In the merging-execution zone, the RCU computes the optimal sequence of merging and merging time at each of the four merging points. Based on this, the vehicles decide their required acceleration for smooth and safe merging at the roundabout.

### Traffic Flow Modeling

For vehicle *i* and time *t*, the position and velocity can be calculated using the kinematic equations as $y_{i}\left(t+1\right)=y_{i}\left(t\right)+v_{i}\left(t\right)\Delta t+0.5a_{i}\left(t\right)\Delta t^{2}$ and $v_{i}\left(t+1\right)=v_{i}\left(t\right)+a_{i}\left(t\right)\Delta t$, where $y_{i}$, $v_{i}$, and $a_{i}$ are the position, velocity, and input acceleration, respectively, and Δt is the discrete time step. The controller of vehicle *i* uses the information of its preceding vehicle i−1 to decide a safe control acceleration $a_{i}$ as

(1) $$a_{i}\left(t\right)=f\left(y_{i}\left(t\right),v_{i}\left(t\right),y_{i-1}\left(t\right),v_{i-1}\left(t\right),\nu_{i}^{r}\left(t\right)\right),$$

where f(·) is the driving decision function (which could possibly be an adaptive cruise control (ACC) or a car-following model), and $\nu_{i}^{r}$ is the target (recommended) speed, which is generated by the automated vehicle according to the instruction given by the RCU.

**Figure 2:**
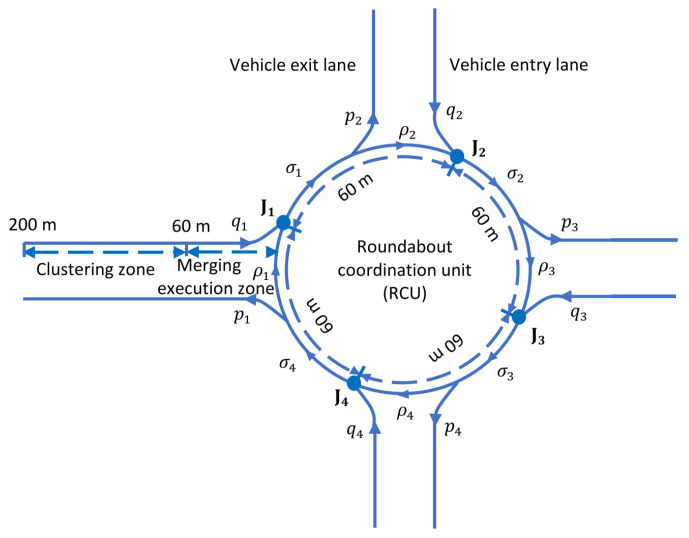
Traffic flow diagram (TFD) of a single-lane four-legged roundabout.

The traffic flow volumes (in veh/min) of the four-legged roundabout can be given by the entry flows $q_{\kappa }$, circulating flows $\rho_{\kappa }$, merged flows $\sigma_{\kappa }$, and exit flows $p_{\kappa }$ with respect to each merging point (junction) $J_{\kappa \in \{1,2,3,4\}}$, as shown graphically using the traffic flow diagram (TFD) in Figure 2. The permissible entry flow and circulating flow rates with respect to the number of lanes within a roundabout can be obtained from [31]. The traffic flow at each junction $J_{\kappa }$ is given by the following relationship

(2) $$J_{\kappa }:\left[\begin{array}{c} \sigma_{\kappa }=q_{\kappa }+\rho_{\kappa },\\ \rho_{\kappa }=\sigma_{\kappa -1}-p_{\kappa },\;\kappa \in \left\{1,2,3,4\right\}, \end{array}\right.$$

where $\sigma_{0}$ is understood as $\sigma_{4}$. Using (2), the flows $\rho_{\kappa }$ of traffic in the roundabout can be obtained using the measured entry flows $q_{\kappa }$ and the exit flows $p_{\kappa }$. As all vehicles are connected, these flows over a certain time interval, e.g., 1 min, can be directly obtained. Such information of the entry flows $q_{\kappa }$ and circulating flows $\rho_{\kappa }$ is required to determine the necessity of vehicle clustering before entering the merging zone.

## 3. Formulation of Optimization Problem

### 3.1. Higher Level Coordination

A vehicle can simply follow another vehicle on the same lane with a minimum following gap of $f_{g}$ s, and both vehicles can pass over the merging point if there is no vehicle approaching from the other (circulating) lane. However, when a vehicle passes the merging point after or before a vehicle from the other lane, it requires an additional safety merging gap of $m_{g}$ s, i.e., two vehicles from different lanes at the merging point require at least a time gap of $m_{g}$ s. Therefore, a pattern involving only a single vehicle merging between two circulating vehicles may affect the incoming traffic of all the entry lanes, which may slow down the overall traffic flows and reduce the capacity of the roundabout. Considering that fact, a higher level coordination is incorporated into the RCS to direct adjacent vehicles in the clustering zone to form vehicle clusters (or platoons) for smooth merging prior to entering the merging-execution zone. The idea of using different time gaps for a pair of vehicles from the same or conflicting directions is based on the actual traffic behavior at roundabouts [32]. Such a time gap selection is also applicable for CAVs when determining their safe trajectories in the conflict zones (merging junctions) at intersections [33]. The function of the higher level coordination is described in this sub-section.

Initially, the necessity of forming vehicle clusters is determined using the information of traffic flow rates. At any junction $J_{\kappa }$, using the entry flow rate $q_{\kappa }$ (veh/min) and circulating flow rate $\rho_{\kappa }$ (veh/min), the number of vehicles (per minute) that leads a cluster (which corresponds to the number of clusters per minute) is determined by

(3) $$n_{\kappa }=\frac{60-f_{g}\left(q_{\kappa }+\rho_{\kappa }\right)}{m_{g}}.$$

When the total flow ($q_{\kappa }+\rho_{\kappa }$) at a merging point $J_{\kappa }$ surpasses its capacity, implying $n_{\kappa }<1$, all vehicles should form a single vehicle cluster. Such an over-saturated traffic condition may create evolving queues and congestion at the roundabout. From the number of clusters $n_{\kappa }$, we can calculate the (recommended) cluster size $s_{\kappa }$ (the average number of vehicles in the cluster) in the entry flow as $s_{\kappa }=q_{\kappa }/\max(1,n_{\kappa })$. When $s_{\kappa }>1$, it is necessary to form vehicle clusters, and some vehicles will be directed to do so, such that the lower level coordination can facilitate smooth merging and avoid a long queue.

**Figure 3:**
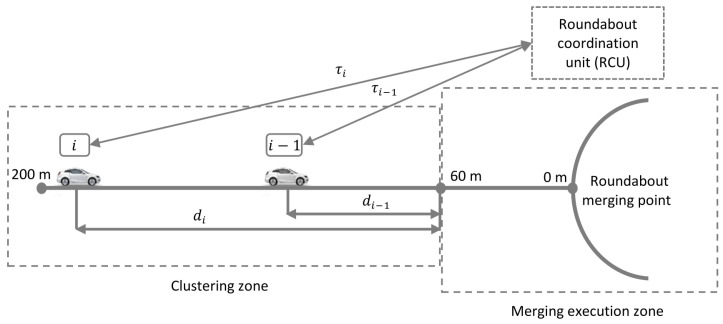
Illustration of the principle to determine the necessity of forming vehicle clusters (higher level coordination). The RCU computes the optimal arrival time of vehicles i−1 and *i*.

Specifically, as illustrated in Figure 3, the RCU coordinates the vehicle clustering by calculating recommended speeds for the vehicles in the entry lane. When vehicle *i* enters the clustering zone, the required possible set of speed of vehicle *i* to reach the end of the clustering zone (at a distance $d_{i}$) are obtained, based on the recommended arrival time of (preceding) vehicle i−1. Then, the RCU calculates the required speed $v_{\mathrm{jci}}$ for vehicle *i* to join a cluster (jc) with vehicle i−1, whilst maintaining a projected time gap $f_{g}$ as $v_{\mathrm{jci}}=d_{i}/\left(\tau_{i-1}+f_{g}\right)$, where $\tau_{i-1}=d_{i-1}/v_{i-1}$ is the estimated time the preceding vehicle arrives at the end of clustering zone. If it is not possible to form a cluster with the preceding vehicle, then vehicle *i* will start a (new) cluster (sc) with its following vehicle (if any), by maintaining a gap $c_{g}=f_{g}+m_{g}$ s with vehicle i−1 (such that a circulating vehicle can pass through), then the corresponding required speed $v_{\mathrm{sci}}$ is calculated as $v_{\mathrm{sci}}=d_{i}/\left(\tau_{i-1}+c_{g}\right)$. If there is no following vehicle, then vehicle *i* will drive at its desired speed $v_{d}$. Using actual speeds $v_{\mathrm{jci}}$ and $v_{\mathrm{sci}}$, as well as the desired speed $v_{d}$, we can calculate $v_{\mathrm{reci}}$, which is the recommended speed for the automated vehicle as

(4) $$v_{\mathrm{reci}}=\left\{\begin{array}{cc} v_{\mathrm{jci}}, & \mathrm{if}\;v_{\mathrm{sci}}\leq v_{\mathrm{jci}}\leq v_{\max},\\ v_{\mathrm{sci}}, & \mathrm{if}\;v_{\mathrm{jci}}\geq v_{\max},\;v_{\mathrm{sci}}\leq v_{\max},\\ v_{d}, & \mathrm{otherwise}, \end{array}\right.$$

where $v_{\max}$ is the maximum allowable cluster speed that can be adjusted according to the desired cluster size $s_{\kappa }$. The local controller of each automated vehicle utilizes this $\nu_{i}^{r}=v_{\mathrm{reci}}$ to compute the acceleration using (1) and safely drive. In this way, the difficulty in merging is reduced, by flexibly coordinating the arrival patterns of automated vehicles. Moreover, such a clustering protocol helps to prevent a long queue at the merging junctions.

### 3.2. Lower Level Coordination

The main factor that causes vehicles to stop prior to entering a roundabout is that they arrive at the merging point almost at the same time of a circulating vehicle. When this happens, the entering vehicles decelerate or stop, increasing fuel consumption and travel time, whilst decreasing average velocity and overall roundabout capacity. Hence, to prevent collision or aggressive braking and minimize idling time, the lower level coordination calculates the optimal time for each vehicle to arrive at the merging point. To mitigate any abrupt and unforeseen changes in traffic flow, the optimal merging algorithm is implemented successively using a receding horizon control approach. Note that we calculate the necessity of forming a vehicle cluster in the higher level coordination based on traffic volume to facilitate merging in the lower level coordination. If vehicles form a cluster in the higher level coordination, they merge in the roundabout as a cluster (which minimizes the waiting time at the merging point) by optimizing the trajectory and merging times of each vehicle in the lower level coordination.

**Figure 4:**
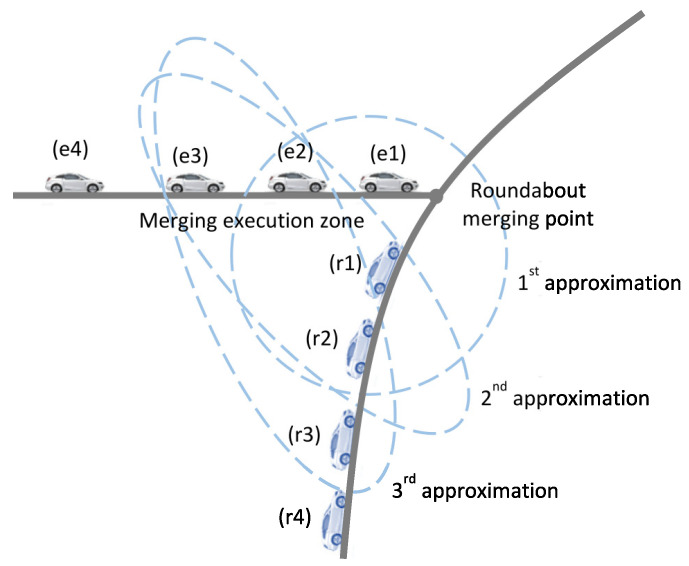
Successive optimization of vehicles (blue dashed regions) in a receding horizon approach in the lower level coordination.

A lower level controller for each junction $J_{\kappa }$ is used to obtain the optimal merging sequence and timings for the vehicles in both the entry and circulating lanes approaching the merging point. The controller considers the vehicles in both the roundabout circulating lane segment (r) and entry lane (e) of the merging-execution zone (see Figure 4). Let E and R be the tuples of vehicles in a sequence on the entry lane (*n* vehicles) and circulating lane (*m* vehicles), respectively, given by $E=\{e_{1},e_{2},\ldots ,e_{n}\}$ and $R=\{r_{1},r_{2},\ldots ,r_{m}\}$, where $e_{1}$ and $r_{1}$ are the vehicles closest to the merging point on the respective lanes. For simplicity, junction index κ is omitted in this description.

Let $v_{\alpha }$ be the speed and $d_{\alpha }$ the distance to the immediate merging point of vehicle α∈E∪R. We assume that vehicle α steadily decreases its speed to merge in the roundabout. Thus, the unrestrained time that a vehicle takes to arrive at the merging point is given by

(5) $$\tau_{\alpha }=\frac{d_{\alpha }}{\frac{1}{2}\left(v_{\alpha }+\psi \right)},$$

where ψ is the allowable merging speed. The optimal merging sequence of the set of vehicles E∪R is obtained, considering the vehicle order constraint that a vehicle is not allowed to overtake on the same lane. Therefore, the search space or the feasible solutions Ω of the merging sequences can be given by a tuple as

(6) $$\Omega =\left\{\begin{array}{c} W=\left(w_{1},w_{2},\ldots ,w_{n+m}\right){|}_{\forall w_{i}\in E\cup R}\\ \;s.t.\;\left[\begin{array}{c} w_{i}\neq w_{j}\;\;\forall i\neq j,\\ \tau_{w_{i}}<\tau_{w_{i+1}},\;\mathrm{for}\;w_{i},w_{i+1}\in E,\\ \tau_{w_{i}}<\tau_{w_{i+1}},\;\mathrm{for}\;w_{i},w_{i+1}\in R, \end{array}\right. \end{array}\right.$$

where, for brevity, the first vehicle in *W* (i.e., $w_{1}$) passes the merging point first, and the rest follows sequentially. According to (6), a vehicle can only be picked once in *W* from E∪R and a following vehicle on the same lane cannot appear before its preceding vehicle.

The objective of the lower level coordination is to obtain the optimal sequence $W^{*}$ of merging by solving

(7) $$\min_{W\in \Omega }J\left(W\right)=\sum_{i=1}^{n+m}\beta_{w_{i}}\tau_{w_{i}}^{*},$$

where $\beta_{w_{i}}$ denotes the weight of individual vehicle and $\tau_{w_{i}}^{*}$ is the optimal passing time of a vehicle $w_{i}$ for a given feasible sequence *W*, which is obtained successively for i=1,2,…,n+m, as $\tau_{w_{i}}^{*}=\max\left(\tau_{w_{i}},\tau_{w_{i-1}}^{*}+\gamma \left(w_{i-1},w_{i}\right)\right)$, where $\tau_{w_{i}}$ is the unrestrained arrival time obtained from (5), and $\gamma (w_{i-1},w_{i})$ denotes the minimum time gap between two vehicles at the merging point given by

(8) $$\gamma \left(w_{i-1},w_{i}\right)=\left\{\begin{array}{cc} \delta_{e}, & \mathrm{if}\;w_{i-1},w_{i}\in E,\\ \delta_{r}, & \mathrm{if}\;w_{i-1},w_{i}\in R,\\ \delta_{m}, & \mathrm{otherwise}, \end{array}\right.$$

where $\delta_{e}$, $\delta_{r}$, and $\delta_{m}$ denote the minimum time gaps between two successive vehicles at the merging point according to their originating lanes.

The above problem falls into the class of combinatorial optimization. We simultaneously solve a combinatorial optimization problem for each roundabout conflict point to obtain the optimal sequence and merging times of vehicles. In this paper, we optimize four vehicles at a time, i.e., two from each lane (n=m=2,m=2) (as illustrated in Figure 4), that makes the optimization problem simple and implementable in real-time. As there are four elements (vehicles) in E∪R, there are 6 feasible solutions in Ω of vehicle sequences considering the vehicle order constraints in the same lane as (6). Hence, the optimization problem (7) is simplified as to pick one of the six possible combinations given in Ω using a brute-force algorithm that systematically evaluates all possible candidates for the solution. When three or more vehicles are present in a lane, the motions of the 3rd vehicle onwards are optimized successively after the leading vehicle has merged, i.e., multiple optimization problems are solved successively. Although our proposed method is able to optimize more than four vehicles at a time, the computation cost will increase considerably. For example, if there are eight vehicles to be coordinated at a time, there will be 70 feasible combinations, which will increase the number of iterations and computation time.

Our approach of using only two vehicles (from each lane) at each optimization is inspired by [11], where only two vehicles approaching an unsignalized intersection from each of six lanes are optimized. Specifically, in [11], since the 3rd vehicle must follow with a safe gap behind the optimized 2nd vehicle, a successive optimization approach can easily coordinate the 3rd vehicle for smooth intersection crossing when it is optimized at the next iteration. Such an approach also works well in the roundabout, as the higher-level coordination usually reduces the possibility of creating a large cluster, unless the traffic is highly congested.

Once the optimal sequence $W^{*}$ of vehicles is determined, each vehicle receives the corresponding recommended merging time $\tau_{w_{i}}^{*}$, and determines the desired speed $\nu_{i}^{r}$ by considering the distance to the merging point. Although the lower level coordination generates a safe merging condition, the coordinated individual vehicles equipped with a local controller must ensure safe merging into the roundabout. For this final check of the safety criterion before merging into the roundabout, a lane change model called minimizing overall braking induced by lane change (MOBIL) is used. Such a lane change happens only at the time of merging. When a vehicle or a cluster of vehicles enters the roundabout, the controller will optimize movements of the next set of vehicles, by repeating the movements of remaining vehicles optimized previously. Thus, the controller successively uses the receding horizon approach to mitigate for inaccurate estimation and prediction, or any changes in vehicle states, while ensuring collision avoidance.

## 4. Simulation Results

**Figure 5:**
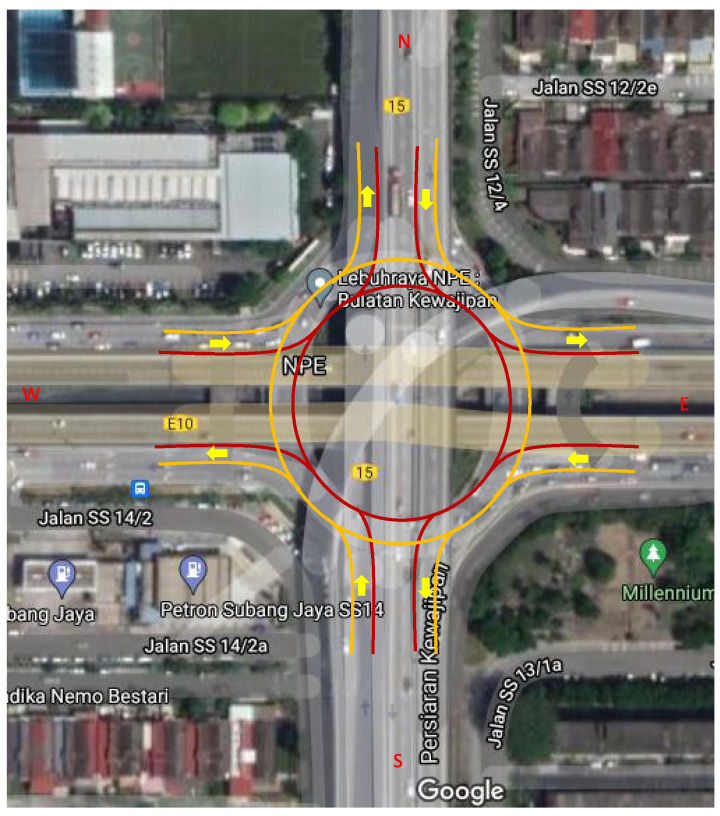
The study roundabout in Subang Jaya, Malaysia taken from the Google map. The outside lane of the roundabout is represented by the orange line and the inner lane is represented by the red line.

To demonstrate the effectiveness of the proposed RCS, we have developed a simulation environment in MATLAB (which has been proven to be mathematically reliable and used to model a variety of real-world scenarios) considering a real roundabout in Subang Jaya, Malaysia as shown in Figure 5. In model building, we have considered a single-lane test-bed for simplicity, while some model parameters are considered according to the real roundabout, e.g., number of junctions, circumference, and traffic flow pattern. The middle area of the roundabout has two lanes, while the four branches each have two lanes and are named North, South, East, and West lanes. The circumference of the roundabout is 240 m long. The traffic flow pattern of this roundabout varies from free flow to congested flow at different times. Although the actual road contains multiple lanes, in the simulation, we only consider the right side lane of the road and the outer lane of the roundabout (indicated by the orange lines in Figure 5). The proposed model can also be applicable for the multi-lane scenarios (where both lanes have the same direction of travel) by incorporating the lane change functionality.

Each entry (approaching) lane is considered to be 200 m long. The clustering zone and the merging execution zone are 140 m and 60 m, respectively. The RCU coverage radius is considered as 250 m (according to IEEE 802.11p). We also assume that communications between vehicles and the RCU are completely reliable. To realize realistic traffic flows, the arrival of vehicles in the entry lanes is given randomly in the simulation using Poisson distribution for different traffic flow rates. Each vehicle is assumed to be 5 m long. For comparison, we first simulate the traditional driving behavior using the intelligent driver model (IDM). Then, we simulate the automated driving behavior as an adaptive cruise control (ACC) system. The vehicle controlled function (1) is used with different values of parameters for human driven and automated vehicles [34]. Note that gap acceptance varies from driver to driver, e.g., gap acceptance of a risky driver is different from a safe driver. In our case, these time gaps for the traditional roundabout system (TRS) are considered as $f_{g}=2$ s and $m_{g}=$ 4 s, according to [35]. Similarly for CAVs, a flow gap of $f_{g}=2$ s and a merge gap of $m_{g}=4$ s are used, subject to coordinated merging with steady speed. The time gaps between two successive vehicles at the merging point are set as $\delta_{e}=\delta_{r}=2$ s and $\delta_{m}=4$ s. The vehicles approach the roundabout, circulate in it, exit independently, and are coordinated only when entering the clustering zone and the merging execution zone. All simulations are run in discrete time with a step size of Δt=0.5 s.

We set the arrival (free-flow) velocity when exiting the clustering zone to be no more than 13.89 m/s (50 km/h) and no less than 10 m/s (36 km/h), in order not to affect the flow of the following traffic. Note that even though the desired velocity is high, the vehicle may move much slower (as the local controller may determine), depending on the motion of the preceding vehicle. The allowable merging speed of entry lane and circulating lane vehicles is set at ψ=30 km/h. To achieve the maximum traffic flow, the circulating vehicles are assigned with higher priority than entry lane vehicles, because delaying the circulating flow will equally affect all lanes, and cause traffic congestion. We set the maximum and minimum velocities of circulating vehicles to be 9.72 m/s (35 km/h) and 5.56 m/s (20 km/h), respectively.

We simulate two traffic flow cases to demonstrate the effectiveness of the proposed RCS for various traffic flow rates (free flow to congested flow near the capacity). In Case 1, all entry lanes have the same traffic flow rates (balanced flow) beginning with 200 veh/h and increased in intervals of 200 veh/h to 1000 veh/h (which is close to the capacity of a single-lane roundabout). The flow rates circulating in the roundabout are assumed to be the same as the entry lane flow rates. In Case 2, the traffic flow rates in the North and South Lanes are twice the flow rates of East and West lanes; the purpose is to create traffic congestion in specific areas, which is a common phenomenon during peak hours. To this end, the traffic flow rate at East and West lanes are initially set at 200 veh/h and the flow rate at North and South lanes are 400 veh/h. Then, the East and West lane flow rates are increased to 600 veh/h in increments of 100 veh/h, while the North and South lane flow rates are doubled. Firstly, the simulations are run to observe the performance of TRS, where all vehicles are driven by humans, with dynamics represented by the intelligent driver model representing function (1) to decide acceleration, and the lane change model called MOBIL is used to execute safe merging. Then, simulations are conducted using the RCS proposed in this paper. Note that due to highly dynamic behavior of traffic flows in real-world circumstances, we run the simulation experiment several times with different random seeds, and take the average values to avoid the influence of randomness on the experimental results. Specifically, we simulate the same group of experiments 10 times with various random seeds.

The comparison of simulation results between RCS and TRS is assessed via five performance metrics of traffic flow, namely (i) average traveling time, (ii) average idling time, (iii) average velocity, (iv) average minimum velocity, and (v) average fuel consumption. The traveling time is the total time taken by vehicles to traverse the roundabout, and the idling time is the total time spent by vehicles to stop and wait at the roundabout junctions. The average velocity is the sum of velocities of all vehicles, divided by the number of vehicles throughout the simulation and the average minimum velocity is the average of minimum velocity of the vehicles. The average minimum velocity indicates whether each vehicle needs to decelerate or stop completely before merging. The average fuel consumption is the total fuel consumption divided by the number of vehicles in the network. The fuel consumption of a vehicle is affected by a number of factors, including the vehicle type, properties, weight, engine size, and power train system. In this paper, the fuel consumption model (based on polynomial fitting with engine torque-speed characteristics) developed by [36] is used to calculate the fuel consumption of individual vehicles. The fuel consumption model, calibrated for a passenger car with a 1.3 L gasoline engine and the continuously variable transmission (CVT) system, fits exactly the fuel consumption rate given in the specification provided by the manufacturer when the car is tested on a standard 10–15 mode fuel-test driving cycle.

**Figure 6:**
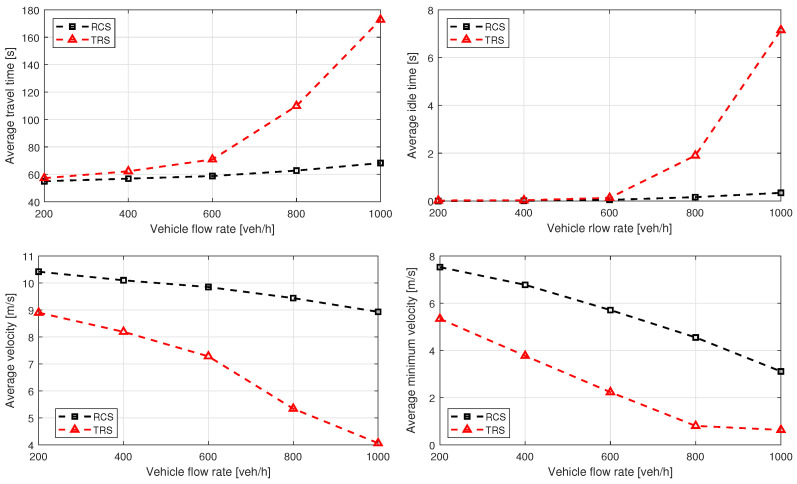
Case 1 performance comparison of roundabout control system (RCS) proposed in this paper and traditional roundabout system (TRS). Each entry lane has the same traffic flow rates and the entry flow rates are equal to the circulating flow rates. The sub-figures show improvements in average traveling time, average idling time, average velocity, and average minimum velocity for the balanced traffic flow.

**Figure 7:**
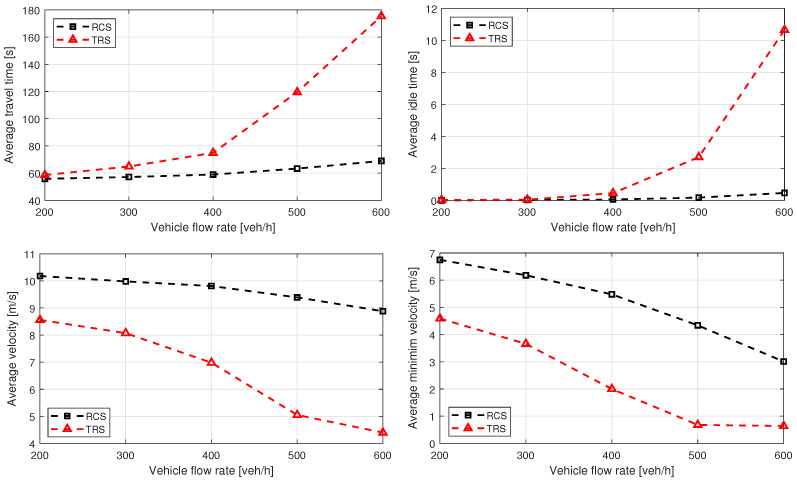
Case 2 performance comparison of roundabout control system (RCS) proposed in this paper and traditional roundabout system (TRS). The North and South lanes have twice the traffic flow of the East and West lanes, and the circulating flow rates are equal to the entry flow rates. The results are plotted against traffic flow of lower density lanes. The sub-figures show improvements in average traveling time, average idling time, average velocity, and average minimum velocity for the unbalanced traffic flow.

**Figure 8:**
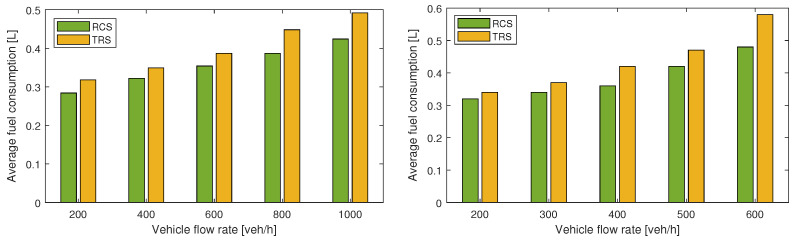
Average fuel consumption of vehicles, Case 1 with balanced traffic flow (**left**) and Case 2 with unbalanced traffic flow (**right**).

Figure 6 and Figure 7 show the simulation results for Cases 1 and 2, respectively, which demonstrate that the proposed RCS causes average traveling and idling times to be significantly lower, compared to the TRS. This is because coordinated vehicles require minimum waiting time before entering the roundabout. However, there may be a trivial increase of traveling time and idling time, where the coordination of vehicles is not possible due to high density of circulating flow. Moreover, the proposed RCS significantly improves the average velocity and minimum average velocity, because coordinated vehicles do not need to slow down or stop in most of the cases before entering the roundabout, which ensures smooth flow. Figure 8 shows the average fuel consumption of both cases, and it is clear that the proposed RCS outperforms the TRS for different traffic demands particularly, when traffic flow is near capacity. The percentage improvements in average travel time, average velocity, and average fuel consumption for Cases 1 and 2 are summarized in Table 1.

## 5. Discussion

In this paper, we consider 100% penetration rate of CAVs, based on the prediction that the vast majority of vehicles will be changed to autonomous vehicles (AVs) after a few decades. However, it is also important to investigate the influence of vehicle coordination in a mixed traffic environment. We keep this investigation out of the scope of this paper, because there is a big hurdle in incorporating traditional human vehicles. Specifically, modeling the behavior of human-driven vehicles in the mixed traffic environment is not well-established in the literature and how they will interact with AVs at roundabouts would be an interesting research topic. On the other hand, once the behavior of human driven vehicles can be predicted, considering their future trajectories as constraints, it will be possible to apply the proposed framework in a similar way to the mixed traffic environment.

Traditional vehicles usually slow down or come to a complete stop before merging. Therefore, it requires a longer time gap (4 s) for merging to satisfy the safety criteria than a typical car-following gap (2 s) according to real-world observations. We also consider the same gap for CAVs for a fair comparison. Since CAVs are coordinated through RCU, they usually merge smoothly at a steady speed, resulting in a significant improvement in roundabout performance. However, CAVs can merge with a smaller gap (e.g., 2.5 s) without slowing down and speeding up (which requires extra time) due to merging at steady speed. In such cases, the performance of the roundabout can be improved further.

Note that substantial communication delay has an impact on CAVs. Specifically, delay has been found to have a significant impact on CACC vehicles, because vehicles in a platoon run with such a small gap that even a small delay can induce platoon instability [37]. On the other hand, in our study, CAVs follow a usual gap that is much larger than CACC. If the communication delay is small enough (i.e., a fraction of the sampling time considered for vehicle coordination), the impact can be kept negligible. It is reported that delays in CAV communication protocols vary from a few milliseconds to 100 ms [38], and in the future (e.g., with 5G-enabled V2X networks), the communication delay is expected to be even lower. In this study, the computation time of our proposed method is in the order of a few milliseconds, and we consider a sampling time of 500 ms. Hence, even the communication delay is up to 100 ms (i.e., one-fifth of the sampling time), it is not necessary to consider the influence of communication delay. Even if there is an unexpected delay or data loss, the CAV configured with a local controller can assure safe merging. In the worst case, when the communication delay is large, e.g., 0.5 s, the coordination can be done at larger sampling steps, e.g., 1 s, while the local controller can drive the car with continuous action. However, it is important to conduct an intensive study on how the magnitude of communication delay affects roundabout performance, which will be the focus of our future work.

The strategy that we present for roundabouts can be tailored to traditional intersections. Since our proposed method can coordinate the merging on multiple roundabout points, it can be easily implemented in merging scenarios (single merging junction or typical crossroad intersections) on the freeway. Our bi-level approach of finding the optimal combination and trajectory optimization can be a potential alternative to centralized control-based intersection coordination, e.g., [11] with high computational complexity. We will conduct such a study in the future.

## 6. Conclusions

In this paper, we have developed a novel roundabout control system (RCS) for CAVs at a four-leg roundabout. The vehicles are coordinated in a bi-level framework using a roundabout coordination unit (RCU). The higher level coordination forms clusters of vehicles based on traffic flow information before preparing for merging. In the lower level coordination, a combinatorial optimization problem is solved to calculate the target times for individual vehicles to enter the roundabout. Following that, a local controller determines the acceleration of automated vehicles for smooth merging and avoiding collision with circulating vehicles. The roundabout coordination is not affected if any vehicle fails to follow the sequence. The proposed RCS is evaluated using a four-leg roundabout considering various traffic demands, i.e., both balanced and unbalanced traffic flow rates and the performance is compared to the traditional roundabout system (TRS). From the results, it is evident that the proposed RCS yields a significant improvement in fuel consumption, travel time, and average velocity of vehicles for different traffic scenarios. The proposed system can be implemented online, as the computational burden is almost negligible.

The current simulation is considered for a single-lane roundabout that will be extended for multi-lane roundabouts in future work. Also, we will investigate mixed traffic performances at roundabouts for various proportions of CAVs. The proposed scheme can be extended further using distributed model predictive control (MPC) for individual vehicles.

## Figures and Tables

**Table 1:** Performance comparison between RCS and TRS.

	TRS	RCS	Improvement
Case 1:			
Average velocity [km/h]	24.34	35.12	44.28%
Average travel time [s]	94.61	60.32	36.20%
Average fuel consumption [ml]	399	354	11.20%
Case 2:			
Average velocity [km/h]	23.81	34.74	45.90%
Average travel time [s]	98.64	60.82	38.34%
Average fuel consumption [ml]	436	384	11.92%
